# A dosimetric study of photon energy and flattening filter mode combinations in Stereotactic Central Ablative Radiotherapy

**DOI:** 10.1002/acm2.70750

**Published:** 2026-08-16

**Authors:** Jianwei Sun, Binghua Liang, Shiqiang Zhang, Heng Zhang, Shi Wang, Xinye Ni

**Affiliations:** ^1^ Department of Radiation Oncology Wuxi Xishan People's Hospital Wuxi Jiangsu China; ^2^ Department of Radiology Wuxi Xishan District Traditional Chinese Medicine Hospital Wuxi Jiangsu China; ^3^ Department of Radiation Oncology The Second People's Hospital of Changzhou, the Third Affiliated Hospital of Nanjing Medical University Changzhou Jiangsu China; ^4^ Department of Engineering Physics Tsinghua University Beijing China

**Keywords:** ablation volume, energy/flattening filter mode combination, Stereotactic Central Ablative Radiotherapy, treatment safety

## Abstract

**Background:**

In Stereotactic Central Ablative Radiotherapy (SCART), the volume of tumor receiving the full prescribed ablative dose decreases rapidly as the prescription dose increases, posing a critical challenge. To address this issue, this study investigates the dosimetric impact of combining various photon energies and flattening filter modes available on modern linear accelerators (linacs), aiming to provide more options for clinical application.

**Methods:**

Fifty‐one cases of abdominal large‐volume tumors treated with conventional radiotherapy at the Second People's Hospital of Changzhou from January 2021 to August 2023 were retrospectively selected. Plans were created using the Monaco 5.11 treatment planning system. All plans employed volumetric modulated arc therapy (VMAT) with two coplanar 360° arcs (clockwise + counterclockwise). The two arcs used different combinations of photon energies/flattening filter modes: 6 MV & 6 MV, 6 MV & 10 MV, 6 MV & 6 MV flattening‐filter‐free (FFF), 10 MV & 10 MV, 6 MV‐FFF & 6 MV‐FFF, and 6 MV‐FFF & 10 MV, while keeping all other planning parameters consistent.

**Results:**

Compared to the standard 6 MV & 6 MV combination, the 6 MV & 10 MV combination reduced the dose to normal tissues without a statistically significant change in ablation volume. The 6 MV & 6 MV‐FFF combination yielded significantly larger ablation volumes, albeit with a statistically significant increase in normal tissue dose, which remained within clinically acceptable limits. The 6 MV‐FFF & 6 MV‐FFF combination also yielded a significantly larger ablation volume, but the increase in normal tissue dose was not statistically significant. The 10 MV & 10 MV and 6 MV‐FFF & 10 MV combinations reduced normal tissue dose while increasing ablation volume, though the increase was less than that achieved with the 6 MV & 6 MV‐FFF and 6 MV‐FFF & 6 MV‐FFF combinations.

**Conclusion:**

The choice of energy/mode combination in SCART can be tailored to clinical priorities. For paramount organ‐at‐risk sparing, 6 MV & 10 MV is recommended. For maximizing ablation volume, 6 MV‐FFF & 6 MV‐FFF is recommended, followed by 6 MV & 6 MV‐FFF. For a balanced approach, the 6 MV‐FFF & 6 MV‐FFF combination demonstrated the best overall performance, followed by the 6 MV‐FFF & 10 MV combination.

## 1 INTRODUCTION

Spatially Fractionated Radiation Therapy (SFRT),^1^, ^2^ a technique relying on spatially heterogeneous dose distributions to treat tumors, has garnered significant attention and shown remarkable progress in recent years due to its potent tumor control capabilities,^3^, ^4^ positioning it as a highly promising direction in radiation oncology research.^5^, ^6^, ^7^ Building upon the core principles of SFRT and the clinical foundation of Stereotactic Body Radiotherapy (SBRT),^8^, ^9^, ^10^ Yan et al. ^11^ proposed a novel spatially fractionated technique—Stereotactic Central Ablative Radiotherapy (SCART).

SCART is specifically designed for treating large‐volume tumors. Its core concept involves delivering an ablative high dose to the central region of the tumor,^12^ maximizing the volume of this high‐dose region within safe margins, while applying a lower, safe dose to the peripheral tumor region to achieve a balance between maximizing central tumor ablation and minimizing peripheral normal tissue toxicity.^13^ As clinical research on SCART deepens, its limitations have become apparent. Recent Phase I clinical trial results indicate that as the prescribed ablative dose to the central tumor region increases, the volume actually receiving this ablative dose exhibits a rapid decline.^14^ A decrease in ablation volume (AV) implies that a smaller proportion of the tumor receives the intended ablative dose, which may reduce tumor control probability (TCP). Several planning strategies could potentially address this limitation, including modifying arc geometry, adjusting optimization objectives, or altering dose fractionation. However, these approaches have been extensively explored in conventional radiotherapy and are already incorporated in current SCART planning protocols. In contrast, the selection of photon beam energy and flattening‐filter(FF) mode directly influences depth‐dose characteristics, penumbra, and out‐of‐field dose, yet it remains largely unexplored in SCART. Modern linear accelerators are routinely equipped with multiple energies and flattening‐filter‐free (FFF) modes, yet current SCART plans uniformly rely on a single 6 MV flattening‐filtered beam. Therefore, systematically evaluating whether different energy/mode combinations can favorably modulate the ablation volume‐normal tissue trade‐off is of substantial clinical relevance. This represents a pragmatic and underexplored optimization pathway.

Modern medical linear accelerators are commonly equipped with multiple photon beam energies and operating modes, such as the FFF mode.^15^, ^16^, ^17^ However, existing SCART treatment plans are predominantly based on the nominal 6 MV x‐ray beam.^18^ Research on utilizing other available energies and modes remains unexplored. The impact of combining different beam energies and modes on SCART plans is not yet clear.^19^ However, conventional 6 MV FF beams have inherent physical limitations, including a relatively flat dose profile with less central dose buildup and a shallower dose fall‐off, which restrict the achievable high‐dose core volume deep within large tumors. Higher‐energy (10 MV) or FFF beams may overcome these limitations by offering deeper penetration, sharper penumbra, or a more conical dose profile with higher central dose.

This study, grounded in SCART theory and aiming to enhance the central ablative dose contribution, increase the probability of triggering desired biological effects, and enlarge the high‐dose conformal volume, systematically designs SCART plans using various energy/mode combinations available on a clinical linear accelerator. By comparing core dosimetric parameters of these combination plans against the standard 6 MV‐only plan, this study evaluates the merits of different combinations regarding key metrics like AV. The findings aim to provide a theoretical basis for the rational selection of beam parameters in clinical SCART treatment, ultimately optimizing therapeutic outcomes for patients.

## 2 MATERIALS AND METHODS

### 2.1 Patient selection

Fifty‐one patients with abdominal large‐volume tumors who completed radiotherapy between January 2021 and August 2023 at the Department of Radiation Oncology, the Second People's Hospital of Changzhou, were retrospectively selected for this planning study. Tumor volume was defined as “large” per prior SCART research:^14^ a minimal diameter > 5 cm on any axial image. The primary tumor locations were the liver (*n* = 16), pancreas (*n* = 13), stomach (*n* = 6), intestine (*n* = 6) and other abdominal sites (*n* = 10).

The mean GTV volume was 853.03 ± 519.26 cm^3^ (median: 605.09 cm^3^), with standard deviation (SD) provided. The corresponding mean STV volume was 94.87 ± 62.55 cm^3^ (median: 66.11 cm^3^). The large standard deviations reflect substantial inter‐patient anatomical and dosimetric heterogeneity, typical for clinical cohorts of large‐volume abdominal tumors.

### 2.2 Target delineation and treatment planning

**FIGURE 1:**
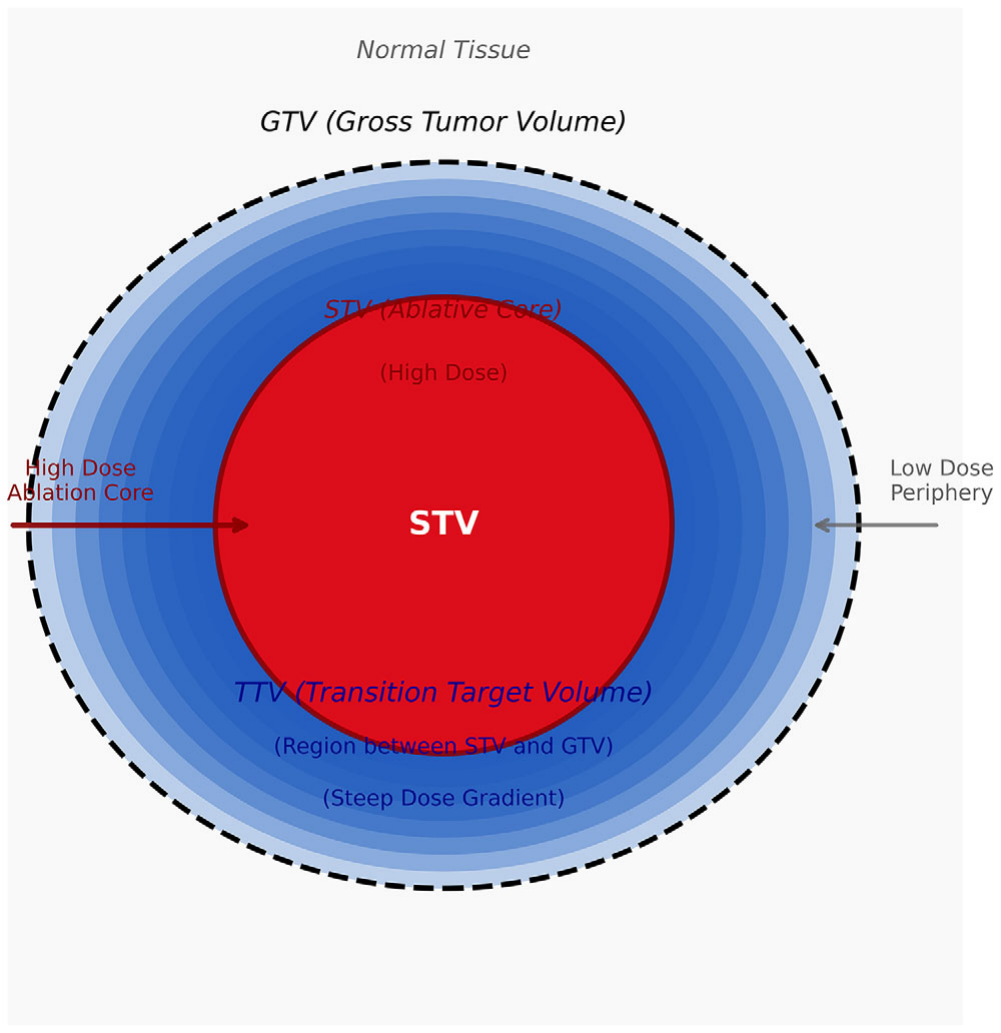
Schematic illustration of target volume definitions in SCART. The gross tumor volume (GTV, dashed boundary) encompasses the entire tumor. The central SCART planning target volume (STV, red core) receives the ablative high dose. The transition target volume (TTV, gradient zone) represents the region between STV and GTV characterized by a steep dose gradient. Normal tissue is shown in light gray. This spatially fractionated dose distribution aims to achieve a balance between central tumor ablation and peripheral normal tissue sparing.

Based on CT and/or MR images, the Gross Tumor Volume (GTV) was delineated as the visible tumor mass, consistent with conventional radiotherapy practice.^20^ In SCART, the Clinical Target Volume (CTV) coincides with the GTV. The central planning target volume within the GTV, termed the SCART‐PTV (STV), is the primary target for the ablative dose. The region between the STV and GTV boundaries is the Transition Target Volume (TTV), where the steep dose gradient occurs. The STV was delineated to encompass approximately 10% of the GTV volume, balancing ablation scope, treatment safety, and potential low‐dose immunostimulation. It was generated using a CT‐based approach: the GTV was uniformly contracted to 30% of its original radial distance in all directions, followed by slice‐by‐slice manual adjustments to match the GTV shape. Individualized modifications in the superior‐inferior direction were applied based on respiratory motion amplitude. This process ensured that the STV remained centrally located within the GTV and away from adjacent normal tissues. Based on Phase I clinical trial results,^14^ the prescription doses were set at 21 Gy × 3 fractions to the STV and 5 Gy × 3 fractions to the GTV. The spatial dose distribution and target volume definitions of SCART (GTV, STV, and TTV) are illustrated in Figure 1.

### 2.3 Treatment planning and energy combinations

All treatment plans were generated using the Monaco® treatment planning system (version 5.11, Elekta AB, Stockholm, Sweden). A standardized volumetric modulated arc therapy (VMAT) technique was employed for all plans, consisting of two coplanar 360° full arcs (one clockwise, one counterclockwise). The core variable in this study was the photon energy/flattening filter mode assigned to these two arcs.

Six distinct combinations were evaluated:

1. 6 MV & 6 MV: Served as the reference baseline plan.
2. 6 MV & 10 MV
3. 6 MV & 6 MV‐FFF
4. 10 MV & 10 MV
5. 6 MV‐FFF & 6 MV‐FFF
6. 6 MV‐FFF & 10 MV

**FIGURE 2:**
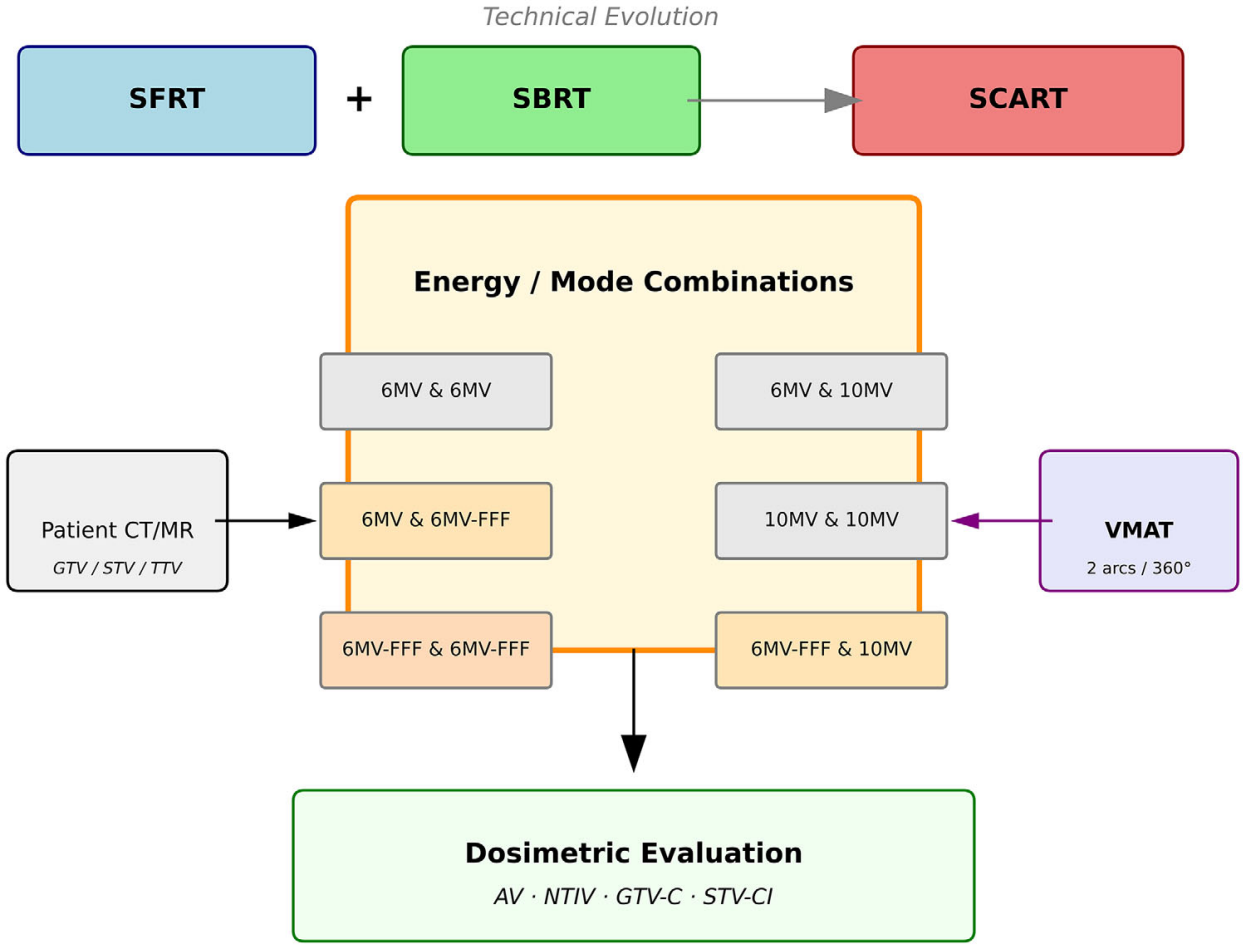
Treatment planning workflow for SCART using different beam energy/flattening filter mode combinations. The technical evolution from SFRT and SBRT to SCART is displayed at the top. The workflow proceeds from left to right: patient CT/MR imaging and target delineation (GTV/STV/TTV) serve as inputs; six different energy/mode combinations are evaluated using VMAT with two coplanar 360° arcs; final dosimetric evaluation is based on four primary endpoints: ablation volume (AV), normal tissue irradiation volume (NTIV), GTV coverage (GTV‐C), and STV conformity index (STV‐CI).

To isolate the effect of the energy/mode combination, all other planning parameters were strictly controlled. This included identical optimization objectives and constraints for all target and organ‐at‐risk volumes, dose calculation algorithm (Monte Carlo), statistical uncertainty, grid size, and arc geometry. The detailed treatment planning workflow, including the six energy/mode combinations and the VMAT technique, is provided in Figure 2.

### 2.4 Plan evaluation and dosimetric endpoints

All generated plans underwent a clinical quality assurance check to ensure they met standard safety and deliverability criteria before comparative analysis.

The primary dosimetric endpoints for comparison were:

1. AV: The volume receiving ≥ 63 Gy (i.e., 100% of the STV prescription).
2. GTV Coverage (GTV‐C): The percentage of the GTV volume receiving ≥ 15 Gy (i.e., 100% of the GTV prescription).
3. Normal Tissue Irradiation Volume (NTIV): The volume of healthy tissue (Body volume minus GTV) receiving ≥ 15 Gy.

AV represents the absolute volume of tumor receiving the full prescribed ablative dose (≥ 63 Gy), which is the primary determinant of tumor control probability (TCP) in SCART. GTV coverage (GTV‐C) reflects the percentage of the entire tumor receiving at least the lower prescription dose (≥ 15 Gy), ensuring that no gross disease is severely underdosed. Normal tissue irradiation volume (NTIV) quantifies the volume of healthy tissue exposed to a non‐negligible dose (≥ 15 Gy), serving as a surrogate for the risk of low‐dose toxicity and secondary malignancy.

Secondary endpoints included the maximum dose (Dmax, defined as the highest point dose within the STV, i.e., the hotspot), and the Homogeneity Index (HI) and Conformity Index (CI) for both the STV and GTV, calculated according to ICRU recommendations. To ensure treatment safety across all abdominal tumor types, organ‐at‐risk (OAR) dose constraints were applied following the Timmerman table for 3‐fraction SBRT.^21^ For each plan, doses to the liver (D30%), kidneys (D30%), spinal cord (Dmax), stomach (Dmax), and intestine (Dmax) were evaluated. The resulting OAR doses for all energy/mode combinations are summarized in Table S1, confirming that all plans met the clinically acceptable OAR dose limits based on the Timmerman table, with a 100% constraint pass rate regardless of tumor location.

### 2.5 TOPSIS‐based multi‐criteria decision analysis

To systematically rank the six energy/mode combinations under different clinical priorities, the Technique for Order Preference by Similarity to Ideal Solution (TOPSIS) was employed. Four dosimetric indicators were selected: ablation volume (AV, benefit type), normal tissue irradiation volume (NTIV, cost type), GTV coverage (GTV‐C, benefit type), and STV conformity index (STV‐CI, benefit type), using their absolute mean values for the TOPSIS analysis. For each of the 51 patients, these four indicators were calculated from the dose‐volume histograms.

The mean values across all patients were then used to construct the decision matrix. Three clinical scenarios were predefined with weight vectors based on expert consensus from three senior radiation oncologists and two medical physicists using the Analytic Hierarchy Process:

OAR‐sparing priority: w = [0.20, 0.50, 0.15, 0.15] for [AV, NTIV, GTV‐C, STV‐CI]

AV priority: w = [0.55, 0.15, 0.15, 0.15]

Balanced scenario: w = [0.35, 0.35, 0.15, 0.15]

The TOPSIS procedure followed standard steps: vector normalization, construction of weighted normalized matrix, determination of ideal and negative‐ideal solutions, calculation of Euclidean distances, and computation of relative closeness (C_i_). The plan with the highest C_i_ under each scenario was considered optimal for that clinical context.

### 2.6 Statistical analysis

Statistical analysis was performed using SPSS Inc., Chicago, IL, USA software (Version 25.0). Continuous data are presented as mean ± standard deviation. As each combination plan was generated on the same patient anatomy, paired sample *t*‐tests were used to compare each of the five experimental plans against the 6 MV & 6 MV baseline plan. A *p*‐value < 0.05 was considered statistically significant.

To reduce the impact of extreme values on statistical estimates, outliers were identified and excluded prior to analysis. Outliers were defined as values below Q1−1.5 × IQR or above Q3 + 1.5 × IQR, where Q1 and Q3 are the first and third quartiles, respectively, and IQR = Q3−Q1. This rule was applied independently to the paired differences (relative changes from the baseline plan) for each dosimetric endpoint and each energy/mode comparison. The number of excluded patients and final sample sizes for each comparison are provided in Tables 1 and 2. To assess the robustness of our findings, a sensitivity analysis was performed including all 51 patients without outlier exclusion, following the same analytical procedures as the primary analysis.

**TABLE 1:** Absolute values and relative changes (vs. baseline) of primary dosimetric endpoints.

Endpoint	6 MV & 6 MV (Baseline)	6 MV & 10 MV	6 MV & 6 MV‐FFF	10 MV & 10 MV	6 MV‐FFF & 6 MV‐FFF	6 MV‐FFF & 10 MV
Ablation Volume (%) (Mean ± SD)	24.50 ± 21.32 (0, *n* = 51)	23.99 ± 22.13 (1, *n* = 50)	31.90 ± 22.33 (3, *n* = 48)	27.15 ± 20.65 (1, *n* = 50)	34.44 ± 20.88 (3, *n* = 48)	29.16 ± 22.20 (2, *n* = 49)
**Δ Ablation Volume (%) (Mean ± SD)**	0 (Ref.)	1.95 ± 72.67	36.68 ± 33.41	30.05 ± 99.95	62.88 ± 70.79	30.51 ± 84.49
*p‐value*	—	0.850	< 0.001	0.039	< 0.001	0.015
GTV Coverage (%) (Mean ± SD)	96.62 ± 20.51 (0, *n* = 51)	96.35 ± 24.06 (4, *n* = 47)	97.14 ± 20.07 (3, *n* = 48)	96.75 ± 20.94 (3, *n* = 48)	97.11 ± 19.13 (4, *n* = 47)	96.53 ± 20.91 (3, *n* = 48)
**Δ GTV Coverage (%) (Mean ± SD)**	0 (Ref.)	−0.45 ± 0.94	0.36 ± 0.71	−0.08 ± 0.57	0.14 ± 0.68	−0.27 ± 0.98
*p‐value*	—	0.002	0.001	0.372	0.182	0.062
NTIV (%) (Mean ± SD)	0.45 ± 0.18 (0, *n* = 51)	0.39 ± 0.17 (0, *n* = 51)	0.50 ± 0.24 (4, *n* = 47)	0.41 ± 0.18 (1, *n* = 50)	0.45 ± 0.19 (4, *n* = 47)	0.40 ± 0.17 (2, *n* = 49)
**Δ NTIV (%) (Mean ± SD)**	0 (Ref.)	−15.24 ± 17.16	9.43 ± 12.29	−9.55 ± 10.26	2.43 ± 8.50	−9.10 ± 16.46
*p‐value*	—	< 0.001	< 0.001	< 0.001	0.062	< 0.001

^#^Abbreviations: Δ, change relative to baseline; NTIV, Normal Tissue Irradiation Volume; AV, Ablation Volume; OAR, Organ‐at‐Risk. “Ref.” denotes the reference baseline value. For each absolute value row, the numbers in parentheses indicate (number of excluded outliers, final sample size n).

**TABLE 2:** Absolute values and relative changes (vs. baseline) of secondary dosimetric endpoints.

Parameter / Plan Combination	6 MV & 6 MV (Baseline)	6 MV & 10 MV	6 MV & 6 MV‐FFF	10 MV & 10 MV	6 MV‐FFF & 6 MV‐FFF	6 MV‐FFF & 10 MV
Max Dose (Hotspot)	7079.62 ± 378.50 (0, *n* = 51)	7023.69 ± 381.77 (3, *n* = 48)	7252.68 ± 418.32 (0, *n* = 51)	7097.00 ± 356.10 (0, *n* = 51)	7363.85 ± 404.78 (0, *n* = 51)	7174.46 ± 415.25 (1, *n* = 50)
**Δ Max Dose (Hotspot) (%)**	0 (Ref.)	−0.60 ± 2.23	2.45 ± 2.58	0.31 ± 3.26	4.05 ± 2.96	1.37 ± 3.08
*p‐value*	—	0.0668	< 0.001	0.4983	< 0.001	0.0028
STV HI	1.56 ± 0.14 (0, *n* = 51)	1.56 ± 0.14 (4, *n* = 47)	1.56 ± 0.14 (3, *n* = 48)	1.55 ± 0.14 (4, *n* = 47)	1.57 ± 0.14 (2, *n* = 49)	1.57 ± 0.14 (1, *n* = 50)
**Δ STV HI (%)**	0 (Ref.)	−0.27 ± 1.79	0.21 ± 2.07	−0.45 ± 2.01	1.00 ± 3.24	0.73 ± 2.97
*p‐value*	—	0.3247	0.4931	0.1357	0.0386	0.0926
STV CI	0.22 ± 0.20 (0, *n* = 51)	0.22 ± 0.21 (2, *n* = 49)	0.30 ± 0.21 (3, *n* = 48)	0.26 ± 0.20 (4, *n* = 47)	0.33 ± 0.19 (3, *n* = 48)	0.30 ± 0.20 (4, *n* = 47)
**Δ STV CI (%)**	0 (Ref.)	−10.16 ± 39.20	32.84 ± 26.09	11.66 ± 35.95	58.92 ± 54.48	20.33 ± 38.19
*p‐value*	—	0.0790	< 0.001	0.0371	< 0.001	0.0013
GTV HI	3.62 ± 0.21 (0, *n* = 51)	3.66 ± 0.22 (1, *n* = 50)	3.67 ± 0.20 (1, *n* = 50)	3.67 ± 0.23 (2, *n* = 49)	3.70 ± 0.23 (1, *n* = 50)	3.71 ± 0.23 (2, *n* = 49)
**Δ GTV HI (%)**	0 (Ref.)	0.88 ± 1.79	1.13 ± 1.45	0.98 ± 2.00	2.29 ± 1.40	1.99 ± 1.67
*p‐value*	—	0.0011	< 0.001	0.0012	< 0.001	< 0.001
GTV CI	0.85 ± 0.04 (0, *n* = 51)	0.86 ± 0.03 (4, *n* = 47)	0.85 ± 0.04 (3, *n* = 48)	0.87 ± 0.03 (1, *n* = 50)	0.85 ± 0.04 (1, *n* = 50)	0.86 ± 0.04 (3, *n* = 48)
**Δ GTV CI (%)**	0 (Ref.)	0.49 ± 1.02	−0.60 ± 1.05	0.99 ± 0.96	−0.18 ± 1.26	0.39 ± 1.02
*p‐value*	—	0.0029	< 0.001	< 0.001	0.3112	0.0173

^#^Abbreviations: Δ, change relative to baseline; HI, Homogeneity Index; CI, Conformity Index. The statistically significant increase in GTV HI (i.e., decreased homogeneity) is consistent with the spatially heterogeneous dose distribution intended in SCART and does not indicate plan degradation. For each absolute value row, the numbers in parentheses indicate (number of excluded outliers, final sample size *n*).

## 3 RESULTS

### 3.1 Comparison of primary endpoints against baseline

**FIGURE 3:**
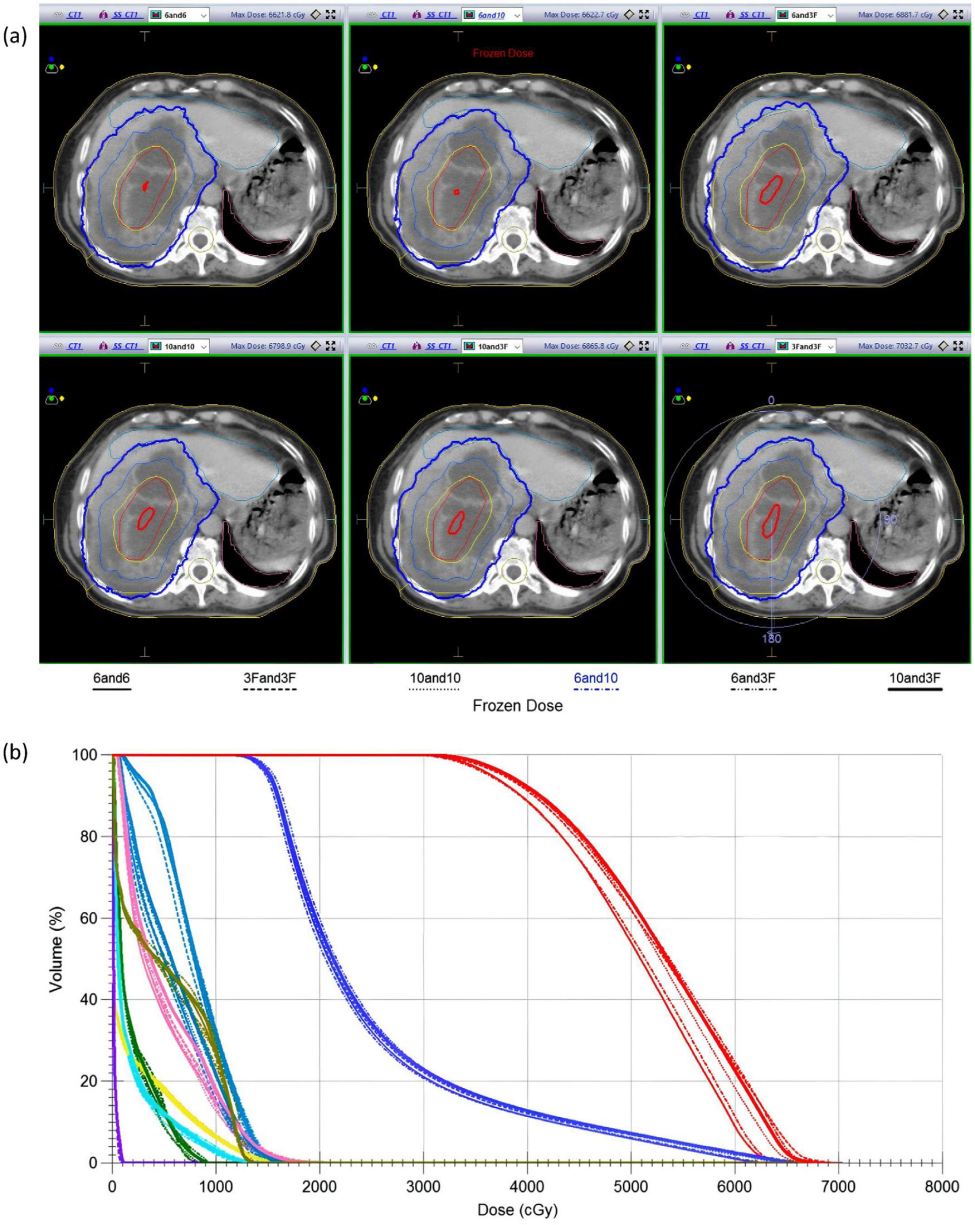
Representative dosimetric comparison. (a) Representative dose distributions of different energy/mode combinations. The 63 Gy isodose line (thick red line) indicates the ablation volume within the STV. The 15 Gy isodose line (thick blue line) represents the peripheral dose covering the GTV. (b) Dose‐volume histograms (DVH) comparing all six energy/mode combinations for the STV, GTV, and key organs‐at‐risk. Different colors represent different structures: red for STV, royal blue for GTV, yellow for body‐GTV, sky blue for heart, purple for intestine, green for left kidney, light blue for right kidney, dark blue for liver, pink for lung, and tan for spinal cord. Within each color, different line styles (solid, dashed, dotted, dash‐dot, etc.) represent the six energy/mode combinations: 6 MV & 6 MV, 6 MV & 10 MV, 6 MV & 6 MV‐FFF, 10 MV & 10 MV, 6 MV‐FFF & 6 MV‐FFF, and 6 MV‐FFF & 10 MV.

The 6 MV & 6 MV combination served as the baseline for all comparisons. A representative dose distribution and plan comparison are shown in Figure 3.

AV: The 6 MV & 10 MV combination did not significantly change the AV compared to baseline (*p* = 0.850). All other combinations significantly increased the AV: 6 MV & 6 MV‐FFF (+ 36.68%, *p* < 0.001), 10 MV & 10 MV (+ 30.05%, *p* = 0.039), 6 MV‐FFF & 6 MV‐FFF (+ 62.88%, *p* < 0.001), and 6 MV‐FFF & 10 MV (+ 30.51%, *p* = 0.015).

GTV Coverage (GTV‐C): The GTV‐C was significantly decreased by the 6 MV & 10 MV combination (−0.45%, *p* = 0.002). The 6 MV & 6 MV‐FFF combination showed a modest increase in GTV‐C (+ 0.36%, *p* = 0.001), and the difference was statistically significant. The changes in GTV‐C for the 10 MV & 10 MV, 6 MV‐FFF & 6 MV‐FFF, and 6 MV‐FFF & 10 MV combinations were not statistically significant.

NTIV: The NTIV was significantly reduced by the 6 MV & 10 MV (−15.24%, *p* < 0.001), 10 MV & 10 MV (−9.55%, *p* < 0.001), and 6 MV‐FFF & 10 MV (−9.10%, *p* < 0.001) combinations. Conversely, the NTIV increased with the 6 MV & 6 MV‐FFF combination (+ 9.43%, *p* < 0.001), while the change with the 6 MV‐FFF & 6 MV‐FFF combination was not statistically significant (+ 2.43%, *p* = 0.062).

A comprehensive summary of the comparisons for these primary endpoints is provided in Table 1.

### 3.2 Analysis of secondary endpoints

The analysis of secondary plan quality indices is detailed in Table 2. Key findings include:

Hotspot (Dmax): A significant increase was observed for plans involving 6 MV‐FFF (6 MV & 6 MV‐FFF, 6 MV‐FFF & 6 MV‐FFF, and 6 MV‐FFF & 10 MV).

STV Conformity (CI): All combinations except 6 MV & 10 MV showed significantly improved STV CI compared to baseline, with the 6 MV‐FFF & 6 MV‐FFF combination showing the greatest improvement (+ 58.92%, *p* < 0.001).

GTV Homogeneity (HI): All experimental combinations showed a statistically significant increase in GTV HI compared to the baseline.

### 3.3 TOPSIS‐based multi‐criteria ranking

To provide a quantitative and transparent ranking of the six energy/mode combinations under different clinical priorities, a TOPSIS‐based multi‐criteria decision analysis was performed using four dosimetric indicators (AV, NTIV, GTV‐C, and STV‐CI) with weight vectors derived from expert consensus.

As shown in Table 3, the optimal combination varied by clinical priority. Under the OAR‐sparing priority, the 6 MV & 10 MV combination achieved the highest relative closeness (C_i_ = 0.6425). Under the AV priority, the 6 MV‐FFF & 6 MV‐FFF combination ranked first (C_i_ = 0.7241). For the balanced scenario, the 6 MV‐FFF & 6 MV‐FFF combination again outperformed all others (C_i_ = 0.6272), followed by the 6 MV‐FFF & 10 MV combination (C_i_ = 0.5659).

**TABLE 3:** TOPSIS‐based ranking of six energy/mode combinations under three clinical priority scenarios.

Plan Combination	OAR‐Sparing Priority	Ablation Volume Priority	Balanced Scenario
6 MV & 6 MV	0.4066 (5)	0.3559 (5)	0.3624 (6)
6 MV & 10 MV	**0.6425 (1)**	0.3296 (6)	0.4787 (5)
6 MV & 6 MV‐FFF	0.3207 (6)	0.6372 (2)	0.5250 (4)
10 MV & 10 MV	0.5787 (2)	0.4865 (4)	0.5306 (3)
6 MV‐FFF & 6 MV‐FFF	0.4724 (4)	**0.7241 (1)**	**0.6272 (1)**
6 MV‐FFF & 10 MV	0.5454 (3)	0.5583 (3)	0.5659 (2)

^#^Data are presented as relative closeness (C_i_) with rank in parentheses. Higher C_i_ indicates better performance. The optimal combination for each scenario is shown in bold.

Sensitivity analysis including all 51 patients without outlier exclusion confirmed that the rankings remained largely consistent with the primary analysis across all three clinical scenarios. Only minor lower‐rank shifts (interchange between the 5th and 6th positions) were observed in the OAR‐sparing and AV priority scenarios, while the ranking for the balanced scenario remained completely unchanged. These results confirm the robustness of our main conclusions. The detailed TOPSIS rankings for this sensitivity analysis are provided in Table S2.

## 4 DISCUSSION

To our knowledge, this is the first dosimetric study to systematically evaluate the impact of combined photon energy and flattening filter modes in SCART planning for large‐volume tumors. Our findings demonstrate that moving beyond the conventional single‐energy paradigm offers a flexible toolkit to modulate the fundamental SCART trade‐off between AV and normal tissue exposure.

The clinical rationale for preserving or expanding the AV in SCART stems from both radiobiological principles and clinical observations. As proposed by Yan et al. and subsequently evaluated in the phase I trial by Yang et al.,^14^ SCART is designed to deliver an ablative high dose to the central tumor region while maintaining a lower, peripheral dose. The central hypothesis is that a larger volume receiving the full ablative dose may increase the likelihood of immunogenic cell death and abscopal responses, which are considered key to systemic antitumor effects. However, Yang et al. found that as the prescribed central dose increased, the achievable AV decreased rapidly, limiting the potential therapeutic benefit of dose escalation. Therefore, identifying beam parameter combinations that preserve or enlarge AV under high prescription doses is clinically relevant, as it may maximize tumor core destruction without exceeding normal tissue tolerance. Conversely, reducing the NTIV directly improves treatment safety by minimizing low‐dose exposure to adjacent organs. In this context, the observed improvements in AV or NTIV with specific energy/mode combinations provide a dosimetric foundation for enhancing both efficacy and safety in SCART. Among the six combinations tested, distinct priorities emerged.

The 6 MV & 10 MV combination was optimal for OAR‐sparing, significantly reducing NTIV by over 15% without compromising AV. This is likely attributable to the higher penetration of the 10 MV beam, which reduces the low‐dose tail outside the field edge, lowers the integral dose to normal tissues, and concentrates the exit dose distribution for deep‐seated tumors[16]. In contrast, when prioritizing AV, the incorporation of 6 MV‐FFF beams—specifically in the 6 MV & 6 MV‐FFF and 6 MV‐FFF & 6 MV‐FFF combinations—demonstrated excellent performance, increasing AV by up to 63%. The higher dose rate and lack of flattening filter in FFF beams lead to a more conical dose profile with a higher central dose, which appears advantageous for building the compact, high‐dose core required in SCART.^22^, ^23^ However, this benefit comes at the cost of increased low‐dose spread to normal tissues, a well‐known characteristic of FFF beams due to reduced head scatter.^24^

The 10 MV & 10 MV and 6 MV‐FFF & 10 MV combinations both reduced NTIV while providing significant (30.05% and 30.51%, respectively) gains in AV. However, the TOPSIS analysis revealed that under a balanced clinical scenario, the 6 MV‐FFF & 6 MV‐FFF combination outperformed both, achieving the highest relative closeness (C_i_ = 0.6272). The 6 MV‐FFF & 10 MV combination ranked second (C_i_ = 0.5659), suggesting its role may be more suited to OAR‐sparing contexts where moderate ablation gain is acceptable. These results align with and extend previous work on mixed‐energy planning in conventional IMRT for other sites, which also found modest but valuable improvements in dose gradients and organ sparing.^25^, ^26^, ^27^

The observed improvement in STV conformity with FFF combinations is a noteworthy secondary benefit. The sharper dose gradient of FFF beams likely enhances the ability to conform the high‐dose region to the centrally located STV, potentially improving therapeutic precision. The observed increase in GTV HI reflects the intended heterogeneous dose distribution of SCART, where a high‐dose core is delivered to the STV while a lower dose is maintained in the periphery. This finding supports the successful realization of the SCART dose profile rather than indicating a deterioration in plan quality.

Our study has several limitations. First, it is a retrospective planning study; the clinical benefits of these dosimetric advantages require validation in prospective trials. Second, our analysis was constrained to the energies (6, 10 MV) and modes available on our specific linear accelerator platform. Investigating other energies (e.g., 15 MV) or the FFF mode for 10 MV could reveal further optima. Third, the plan optimization was performed using a single Monaco Treatment Planning System (TPS). While the core physics principles are generalizable, the absolute magnitude of differences might vary with other dose calculation algorithms or optimization engines. Fourth, although we strictly controlled all planning parameters except the energy/mode combination to isolate its dosimetric effect, this controlled approach may not fully reflect clinical practice. In routine planning, objectives, constraints, and optimization trade‐offs are typically adjusted according to beam characteristics and patient anatomy. We therefore acknowledge that the absolute magnitude of the reported differences may vary when each energy/mode combination is independently re‐optimized in a clinically realistic setting. Nevertheless, the observed directional trends–particularly the superiority of 6 MV‐FFF & 6 MV‐FFF for maximizing AV and 6 MV & 10 MV for OAR‐sparing–are rooted in fundamental beam physics (depth‐dose characteristics, penumbra, and head scatter) and are expected to persist after clinically realistic re‐optimization. Finally, our findings are derived exclusively from abdominal large‐volume tumors. Due to the limited sample size within each tumor site subgroup (liver, pancreas, stomach, and intestine), we did not perform site‐specific subgroup analyses or establish separate dose constraints for each tumor type. Instead, we applied unified OAR constraints based on the Timmerman table (Table S1), which all plans met. Future studies with larger, site‐specific cohorts are needed to validate the generalizability of our findings and to develop location‐specific dose guidelines.

As linac and TPS technology advances, seamless switching and combination of different energies and modes will become more streamlined, paving the way for superior plan design. Furthermore, the future may see hybrid treatments combining various radiation modalities (e.g., protons, ions) or ultra‐high dose rate (FLASH) techniques, leveraging their unique advantages to achieve plans with superior tumor kill and normal tissue protection.

## 5 CONCLUSION

This dosimetric study suggests that the strategic combination of different photon beam energies and flattening filter modes may favorably modify the dose distribution in SCART. Selective adjustment of these parameters appears to improve central ablative volume while maintaining target coverage and respecting peripheral normal tissue constraints. Based on these preliminary findings, the following considerations may serve as a hypothesis‐generating framework for SCART of large‐volume tumors:

1. For cases where organ‐at‐risk sparing is a primary concern, the 6 MV & 10 MV combination may be considered.
2. If maximizing AV is the priority, the 6 MV‐FFF & 6 MV‐FFF combination may be preferable, with the 6 MV & 6 MV‐FFF combination as an alternative.
3. For a balanced approach that aims to address both endpoints, the 6 MV‐FFF & 6 MV‐FFF combination may be a reasonable option, with the 6 MV‐FFF & 10 MV combination as an alternative.

These findings provide a preliminary dosimetric framework for priority‐based personalized SCART planning, which may offer the potential to improve both the efficacy and safety of this technique.

## AUTHOR CONTRIBUTIONS

Jianwei Sun and Binghua Liang wrote the main manuscript text and prepared the original draft. Jianwei Sun, Binghua Liang, Shiqiang Zhang, and Heng Zhang performed data curation and validation. Jianwei Sun and Shi Wang contributed to conceptualization and formal analysis. Shi Wang and Xinye Ni reviewed and edited the manuscript. Xinye Ni supervised the project and acquired funding. All co‐authors have reviewed and approved the final manuscript.

## CONFLICT OF INTEREST STATEMENT

The authors declare no conflicts of interest.

## ETHICS STATEMENT

This study was conducted in accordance with the Declaration of Helsinki and was approved by the Ethics Committee of the Second People's Hospital of Changzhou (Approval No. [2024]KY210‐01). The requirement for informed consent was waived due to the retrospective nature of the study.

## Supporting information



Supporting information: acm270750‐sup‐0001‐tableS1.docx

Supporting information: acm270750‐sup‐0002‐tableS2.docx

## Data Availability

The data that support the findings of this study are available from the corresponding author upon reasonable request.
