# Supplementary material for: A dosimetric study of photon energy and flattening filter mode combinations in Stereotactic Central Ablative Radiotherapy
Source: J Appl Clin Med Phys. 2026 Aug 16;27(8):e70750. doi: 10.1002/acm2.70750 (PMC13478369; doi:10.1002/acm2.70750)
Supplement: Supplementary file 1 [file ACM2-27-e70750-s001.docx]

| **Plan Combination** | **Parameter** | **Liver D30% (cGy)** | **Kidney（L） D30% (cGy)** | **Kidney（R） D30% (cGy)** | **Spinal Dmax (cGy)** | **Stomach Dmax (cGy)** | **Intestine Dmax(cGy)** |
| --- | --- | --- | --- | --- | --- | --- | --- |
| **6 MV & 6 MV (Baseline)** | Mean ± SD | 763.6 ± 347.1 | 705.8 ± 312.2 | 708.5 ± 385 | 1085.4 ± 219.2 | 1210.7 ± 518.5 | 1533.3 ± 297 |
|  | max | 1350 | 1126 | 1093 | 1376.5 | 1563 | 2217.5 |
| **6 MV & 10 MV** | Mean ± SD | 752.3 ± 341.8 | 714.4 ± 294.7 | 706.9 ± 378.7 | 1074.3 ± 203.9 | 1199.6 ± 501 | 1491.5 ± 324.8 |
|  | max | 1344 | 1093 | 1069 | 1372 | 1554 | 2277.5 |
| **6 MV & 6 MV-FFF** | Mean ± SD | 774.5 ± 351.9 | 725.8 ± 305.5 | 726.3 ± 397.5 | 1091.8 ± 193.3 | 1224.3 ± 526.7 | 1538.5 ± 296.4 |
|  | max | 1365 | 1085 | 1104 | 1413 | 1568 | 2190.5 |
| **10 MV & 10 MV** | Mean ± SD | 752.3 ± 343.4 | 703.7 ± 293.5 | 704.5 ± 378.8 | 1071.6 ± 222.3 | 1218.9 ± 519.1 | 1502.8 ± 313.2 |
|  | max | 1336 | 1076 | 1062 | 1398.5 | 1569 | 2196.5 |
| **6 MV-FFF & 6 MV-FFF** | Mean ± SD | 754.2 ± 349.7 | 711 ± 302.3 | 707 ± 390.6 | 1096.2 ± 193.9 | 1225.7 ± 536.1 | 1520.6 ± 310.6 |
|  | max | 1357 | 1081 | 1082 | 1409.5 | 1598 | 2222.5 |
| **6 MV-FFF & 10 MV** | Mean ± SD | 753.5 ± 336.4 | 700.2 ± 289.4 | 690.2 ± 370.3 | 1072.2 ± 191.3 | 1195 ± 521.3 | 1496.9 ± 329.1 |
|  | max | 1372 | 1078 | 1066 | 1366.5 | 1568 | 2204.5 |

Table S1. Organ-at-risk doses for different energy/mode combinations.

\# SD, Standard Deviation; Dmax, Maximum Dose;D30%, Dose to 30% of the volume. To ensure treatment safety, the dose constraints for the liver and kidneys were evaluated using D30%, which requires that at least 70% of the organ volume receive a dose of less than 17.7 Gy (based on the Timmerman table, updated August 2021: liver D33% < 17.7 Gy). The same criterion was applied to both kidneys. All plans satisfied the clinically acceptable OAR dose limits based on the Timmerman table, with a 100% constraint pass rate.
