# Supplementary material for: A dosimetric study of photon energy and flattening filter mode combinations in Stereotactic Central Ablative Radiotherapy
Source: J Appl Clin Med Phys. 2026 Aug 16;27(8):e70750. doi: 10.1002/acm2.70750 (PMC13478369; doi:10.1002/acm2.70750)
Supplement: Supplementary file 2 [file ACM2-27-e70750-s002.docx]

| **Plan Combination** | **OAR-Sparing Priority** | **Ablation Volume Priority** | **Balanced Scenario** |
| --- | --- | --- | --- |
| 6 MV & 6 MV | 0.2947 (6) | 0.2886 (6) | 0.3136 (6) |
| 6 MV & 10 MV | **0.6430 (1)** | 0.3282 (5) | 0.4878 (5) |
| 6 MV & 6 MV-FFF | 0.3063 (5) | 0.6487 (2) | 0.5157 (4) |
| 10 MV & 10 MV | 0.5870 (2) | 0.4909 (4) | 0.5235 (3) |
| 6 MV-FFF & 6 MV-FFF | 0.4516 (4) | **0.7258 (1)** | **0.6134 (1)** |
| 6 MV-FFF & 10 MV | 0.5436 (3) | 0.5838 (3) | 0.5919 (2) |

Table S2. Sensitivity analysis: TOPSIS-based ranking of six energy/mode combinations under three clinical priority scenarios (n=51).

\# Data are presented as relative closeness (Cᵢ) with rank in parentheses. Higher Cᵢ indicates better performance. The optimal combination for each scenario is shown in bold.
